# Patient satisfaction with surgical informed consent at Jimma Medical Center, Ethiopia

**DOI:** 10.1186/s12910-022-00841-5

**Published:** 2022-10-25

**Authors:** Tsegaw Biyazin, Ayanos Taye, Yeshitila Belay

**Affiliations:** 1 School of Midwifery, Faculty of Health Science, Institute of Health, Jimma University, P.O. Box 378, Jimma, Ethiopia; 2 School of Nursing, Faculty of Health Science, Institute of Health, Jimma University, P.O. Box 378, Jimma, Ethiopia

**Keywords:** Informed consent, Patient satisfaction, Surgical patients, Jimma

## Abstract

**Background:**

Informed consent is a process in which a healthcare provider obtains permission from an individual prior to surgery. Patient satisfaction with the informed consent process is one of the main indicators of healthcare service quality. This study aimed to assess patient satisfaction with surgical informed consent at Jimma Medical Center, Ethiopia, in 2020.

**Methods:**

A facility-based cross-sectional study was conducted from April 1 to June 30, 2020, at Jimma Medical Center. Face-to-face interviews were conducted using structured questionnaires. A systematic sampling technique was used to select the study participants. The collected data were coded, entered into Epi data version 3.1, and analyzed using SPSS version 25. Bivariate and multivariate regression analyses were performed to determine the association between patient satisfaction and socio-demographic and facility-related factors. In multivariate regression, predictors with a *P*-value of < 0.05 were considered statistically significant.

**Results:**

Totally 372 study participants were interviewed with a response rate of 97.8%. Nearly two-fifths (43%) of patients were satisfied with surgical informed consent. Living in an urban area (AOR: 2.279, 95% CI 1.257–4.131), having current referred history (AOR: 1.856, 95% CI 1.033–3.337), consent form version (AOR: 2.076, 95% CI 1.143–3.773), time spent on the provision of informed consent (AOR: 5.227, 95% CI 2.499–10.936) and having better patient-health providers relationship (AOR: 5.419, 95% CI 3.103–9.464) predictors were positively associated with patient satisfaction.

**Conclusion:**

Patient satisfaction with the surgical informed consent process was relatively low. Therefore, Health care professionals need to emphasize a way of delivering informed consent, patients' needs and obey a standard informed consent to improve patient satisfaction.

## Introduction

Surgical informed consent is one vital part of preoperative care [1–3]. Informed consent is the process by which a healthcare provider obtains permission from an individual before delivering therapy, treatment, or surgery [4]. The process of informed consent involves more than signing a prescribed form [5]. Providing proper surgical informed consent to patients who have undergone surgery makes them satisfied [6–8].

Patient satisfaction is a major indicator of quality of healthcare services [9]. Patient satisfaction is the degree to which a patient meets the expectations of ideal care based on their perception of the actual care received [10, 11]. Patient satisfaction during informed consent increases when written informed consent is provided, combined with verbal informed consent during the preoperative period [12]. Satisfied patients are more likely to obey treatment, take an active role in their care, continue using health care services, voluntarily participate in decision making, and stay within a healthcare provider [13].

Previous studies have reported that the level of patient satisfaction with informed consent varies between countries. The studies conducted in Pakistan, Israel, Netherland, and Switzerland showed that patient satisfaction with surgical informed consent ranged from 49 to 95% [14–17]. In contrast, patient satisfaction with the provision of surgical informed consent in African countries is relatively low. The studies conducted in Rwanda, Botswana, Eritrea, and Hawassa, Ethiopia revealed that the overall patient satisfaction with surgical informed consent ranged 36.9–67.4% [18–21].

patients who were satisfied with their health care services showed greater adherence to treatment plans, fewer hospital readmissions, and greater intention to keep follow-up appointments; patients treated at the hospital with higher patient satisfaction scores experienced lower rates of postoperative mortality, and death after any complication [22].

Different studies revealed that factors affecting patient satisfaction in preoperative care services include, lack of a standard consent form, lack of readiness to deal with urgent medical conditions, overcrowding of the surgical unit, younger age, low literacy level, Patient knowledge and understanding of surgery, patients who underwent elective surgery, patients who had experience with disease or operation, lack health care provider experience with SIC, heavy workload for health care providers, delay in requesting consent, time spent on informed consent provision and patient-doctor relationship [7, 20, 23–30].

Worldwide, different attempts have been made to increase health care quality by improving patient satisfaction. Recent studies have recognized perioperative surgical home (PSH) as a way of working with patients to optimize their condition before surgery, the intraoperative phase, the immediate postoperative phase, and post-discharge [31]. In Ethiopia, efforts have been made to enhance patient satisfaction through informed consent. The effort included; preparing Medico-legal guidelines, incorporating Medical ethics for doctors in the Curriculum, and provision of Compassionate, Respectful, and Caring health care providers are already commenced [32]. However, it doesn’t provide adequate results to enhance patient satisfaction with the surgical procedures. It has been confirmed; that litigation issues for healthcare providers increase over time [33].

Previous studies in Ethiopia have been limited to patient satisfaction with informed consent. Therefore, this study aimed to assess patient satisfaction with the provision of surgical informed consent at Jimma Medical Center, Jimma, Ethiopia.

## Methods

### Study design and setting

This cross-sectional study was conducted at Jimma Medical Center, Jimma, Ethiopia. Jimma Medical Center is a referral hospital in Southwest Ethiopia. It is a teaching tertiary hospital with four major clinical departments including internal medicine, surgery, pediatrics, and gynecology/obstetrics; and other clinical departments, dentistry, ophthalmology, Orthopedics, psychiatry, Reproductive health center, dermatology, and Antiretroviral therapy (ART) clinics. The obstetrics and gynecology ward had 76 beds available for postoperative care. Approximately 1,461 and 900 patients underwent obstetric and gynecological-related surgery within the past six months respectively (previous 6-month report). A total of 787 patients underwent surgery within two months. The study period was from April 1 to June 30, 2020.

### Study participants

All women who underwent obstetric and gynecological surgeries were included in the study. Women who underwent obstetric and gynecological surgeries during the study period were selected as the study population.

### Inclusion and exclusion criteria

Women who underwent Ob-Gyn surgery and those aged ≥ 18 years were included in this study. However, those who were critically ill and had known psychiatric illnesses were excluded.

### Sample size determination and technique

The sample size was determined using the single population proportion formula by considering 62% proportion (P) which took from research conducted in Hawassa [21]; with a 95% confidence interval (1.96); α = 0.05 and 5% marginal of error. By adding a non-response rate of 5%, the final sample size was 380. A systematic sampling technique was employed to select study participants from a total of 787 two-month surgical cases after determining the interval (K value). The k-value was determined by dividing the total two-month surgical case (787) by the final sample size (380) which was approximately 2. The first study participant was selected using a simple random technique from the first two individuals and the second participant was chosen every two intervals from the registration book until the final sample size was reached.

### Data collection tool and procedure

Data were collected using a structured closed-ended questionnaire. The data collection tool was adapted from different studies developed for similar purposes [2, 15]. The questionnaires have six parts. The first part about general socio-demographic characteristics consists of 8 items, the second part deals with patient-related factors consists of 7 items; the third one about service-related factors consists of 8 items, and the fourth part deals with the patient-healthcare relationship consisting of 9 items, adopted from validation of the patient to doctor relationship questionnaire (PDRQ-9) study conducted in German [34], the fifth part deals with patients’ knowledge of surgical informed consent and the last part deals with satisfaction with informed consent, it measured using 10 items with a five-point Likert scale option (ranging from 1 completely dissatisfied to 5 completely satisfied). Two BSc nurses and one MSc nurse were involved in the data collection and supervision respectively. Data were collected using a face-to-face interview.

### Data quality management

The questionnaire was initially prepared in English then translated into the local languages (Afaan Oromo and Amharic) and then translated back into English. Prior to the actual data collection, a pretest was conducted on 5% of the total sample (19 women) at Shenen Gibe Hospital. Appropriate corrections and mandates were made to the questionnaire. The one-day training was provided to the supervisor and data collectors. During the data collection period, the data were checked for completeness and consistency. Any error, ambiguity, incompleteness, or other problems were addressed through communication with data collectors before the beginning of the next day's data collection activities. The reliability of the patient satisfaction tool was confirmed using Cronbach’s Alpha, which was 0.83.

### Data analysis procedure

The collected data were coded and entered into Epi data version 3.1 and exported into SPSS version 25. The Hosmer and Lemeshow test was performed to confirm model fitness. Bivariate and multivariate analyses were performed to compare the satisfaction of surgical informed consent with predictors. In bivariate logistic regression, the variables which had a *P*-value < 0.25 were considered candidate variables for multivariable logistic regression. In multivariable regression, variables with a *P*-value < 0.05 were considered statistically significant with the outcome variable. Finally, the results were narrated using text, tables, figures, and graphs.

### Ethical consideration

Ethical clearance was obtained from Institutional Review Board of Jimma University (IRB Ref No: 000,165/2020). Permission letter was obtained from Jimma Medical Center. The purpose and importance of the study were explained and verbal informed consent was secured. Confidentiality was maintained at all level of the study. Participants’ involvement in the study was on voluntary bases and that they could withdraw at any time if they want. All the information given by the respondents was used for research purposes only.

## Results

### Socio-demographic characteristics

A total of 372 study participants were involved in the study with a response rate of 97.8%. The majority 310 (83.3%) of study respondents were married and 324 (87.1%) were aged < 35 years. The majority 182 (48.9%) were Muslim, and 267 (70.1%) study participants were Oromo. The majority of 242 (65.1%) participants were living in Urban, 168 (45%) were housewives, 291 (78.2%) were literate and nearly half 47.1% had less than 1500 EB monthly income (Table 1).

**Table 1:** Socio-demographic characteristics of study participants at JMC, Ethiopia, 2020 (n = 372)

Variables	Classification	Frequency	Percent (%)
Age	< 35	324	87.1
	> 35	48	12.9
	Total	372	100.0
	Mean and SD	29.5 ± 3.5	
Education status	Illiterate	81	21.8
	Literate	291	78.2
Marital status	Single	50	13.4
	Married	310	83.3
	Divorced/widowed	12	3.2
Religion	Orthodox	123	33.1
	Protestant	64	17.2
	Muslim	182	48.9
	Other^1^	3	0.8
Ethnicity	Oromo	267	71.8
	Amhara	37	9.9
	Tigre	5	1.3
	Gurage	59	15.9
	Other^2^	4	1.1
Occupation	Housewife	168	45.2
	Private employee	33	8.9
	Government employee	68	18.3
	Merchant	29	7.8
	Farmer	56	15.1
	Student	18	4.8
Residence	Urban	242	65.1
	Rural	130	34.9
Monthly income	< 500 ETB	110	29.6
	501–1500 ETB	65	17.5
	1501–2500 ETB	71	19.1
	2501–3500 ETB	43	11.6
	3501–4500 ETB	22	5.9
	> 4501ETB	61	16.4

Other ^1^Catholic ^1^wakefata ^2^Yem, ^2^Dawuro

### Patient-related factors

More than half of the respondents (52.2%) were multipara and three-fourths of study participants (74.5%) had undergone an emergency surgical procedure. The majority of study participants 319 (86%) underwent operation with a reason of pregnancy-related cases followed by 20 (5.4%) gynecological cancers, 12(3.2%) gynecological benign tumors, and 11 (3%) pelvic organ disorders. One hundred three (27%) respondents had a previous history of surgery, of which 60 (58.3%) of them had once a surgical history while the remaining 43 (41.7%) patients had more than one surgical history. A majority (77.1%) of women had poor knowledge, and the remaining one-quarter (22.9%) of them had good knowledge of ward surgical informed consent (Table 2).

**Table 2:** Patient-related factors, Jimma Medical center, Ethiopia, 2020 (n = 372)

Variables	Category	Frequency	Percent
Parity	Null	14	3.8
	Prim-para	160	43.0
	Multipara	198	53.2
Schedule of surgery	Elective/Planned	95	25.5
	Emergency/Unplanned	277	74.5
Reason for the operation	^1^Gynecological benign tumor	12	3.2
	^2^Gynecological cancer	20	5.4
	Fistula	10	2.7
	Related to pregnancy	319	85.8
	^3^Pelvic organ prolapse	11	3.0
Previous medical history	No	330	88.7
	Yes	42	10.3
Previous surgical history	Yes	103	27.7
	No	269	72.3

^1^Fibroma, Myoma, Adenoma, Adenomyosis. Endometriosis, polyps …etc.

^2^Cervical Ca (squamous cell carcinoma or adenocarcinoma), Endometrial Ca, Ovarian Ca….etc.

^3^Cystocele, Urethrocele, Uterine prolapse, Vaginal vault prolapse, Rectocele & Enterocele

### Service-related factors

Two hundred fifty-eight (69.4%) study participants were referred from other health facilities. Nearly two-thirds, 62.9% of women were responding that the consent form was written in their mother tongue. The majority of 302 (81.2%) study participants reported that surgical informed consent was obtained by GP/resident, followed by obstetrician-gynecologists 44 (11.8%) and nurse/midwives 26 (7%). More than half of 208 (56%) respondents reported that they received informed consent counseling immediately before surgery. The consent form was signed by the patients themselves 352 (94.6%) while the remaining 20 (5.4%) were signed by their relatives (Table 3).

**Table 3:** Service-related factors of satisfaction with SIC, Jimma Medical Center, Ethiopia, 2020 (n = 372)

Variables	Category	Frequency	Percent
Referred history	Yes	258	69.4
	No	114	30.6
Written consent form version	Within mother tongue	234	62.9
	Deferred from mother tongue	124	33.3
Consent requested by	Ob-gyn specialist	44	11.8
	General practitioner/resident	302	81.2
	Midwife/nurse	26	7.0
Timing of consent	The day before the date of surgery	65	17.5
	On the day of surgery	87	23.4
	Immediately before surgery	208	55.9
	On the operation table	12	3.2
Time spent to provide informed consent	< 5 min	231	62.1
	5–10 min	76	20.4
	> 10 min	65	17.5
Time spent to decide	< 1 h	343	92.2
	> 1 h	29	7.8
A person signed on informed consent	Self	352	94.6
	Parent/spouse	20	5.4

### Patient- health provider relationship

Women were asked to assess the patient-healthcare providers’ relationship while they provided informed consent. Out of the total interviewed study participants, 179 (48%) women had a good patient health providers relationship.

### Patient satisfaction with surgical informed consent

Women have scored the highest satisfaction value on three satisfaction measurement items; i.e. awareness of the benefit of operation, involvement in the discussion about the operation, and involvement in decision making accounting for 79.1%, 89.5%, and 91.2% respectively (Table 4).

**Table 4:** Satisfaction of respondents with SIC at JMC, Ethiopia, 2020

Items	Completely dissatisfied	Dissatisfied		Neutral	Satisfied	Completely satisfied
The information on an indication of operation	26 (7.0%)	166 (44.6%)	13 (3.5%)		124 (33.3%)	43 (11.6%)
The alternative of operation	28 (7.5%)	89 (23.9%)	163 (43.8%)		71 (19.1%)	21 (5.6%)
The chance to express opinions	25 (6.7%)	223 (59.9%)	12 (3.2%)		89 (23.9%)	23 (6.2%)
The chance to ask questions	22 (5.9%)	210 (56.5%)	16 (4.3%)		95 (25.5%)	29 (7.8%)
The amount of information about the operation	33 (8.9%)	150 (40.3%)	17 (4.6%)		131 (35.2%)	41 (11.0%)
The information was easy to understand	32 (8.6%)	133 (35.8%)	13 (3.5%)		155 (41.7%)	39 (10.5%)
The awareness on the benefit of the operation	9 (2.4%)	49 (13.2%)	20 (5.4%)		210 (56.5%)	84 (22.6%)
The awareness on the risk of operation	10 (2.7%)	90 (24.2%)	156 (41.9%)		56 (15.1%)	60 (16.1%)
Involvement in the discussion about the operation	3 (8%)	23 (6.2%)	13 (3.5%)		166 (44.6%)	167 (44.9%)
Involvement in the decision making on the operation	7 (1.9%)	15 (4.0%)	11 (3.0%)		117 (31.5%)	222 (59.7%)

Women were asked to assess their level of satisfaction with the SICs they had received prior to their surgical procedure on a five-point scale. The findings of this study showed that 160 (43%) respondents were satisfied and the remaining 212 (57%) respondents were dissatisfied with the informed consent provision.

### Factors associated with Satisfaction of informed consent

In bivariate logistic regression, variables with a *P*-value less than 0.25 were considered as candidate variables for multivariate logistic analysis. These variables were residence, marital status, occupation, parity, type of surgery, referred history from another health facility, the version of a written consent form, timing of consent, time taken to provide informed consent, the person signing the consent form, profession who request for the operation, knowledge of SIC and patient to healthcare provider relationships (Table 5). However, in the multivariable regression, candidate variables were entered into the regression using the backward elimination method; and only five predictors included:- residence, referred history from another hospital, Version of a written consent form, time spent while providing SIC, and patient-healthcare provider relationship were statistically significant with patient satisfaction (Table 6).

**Table 5:** Bivariate logistic regression of factors associated with patients’ satisfaction on SIC 2020

Variables	Category	Satisfaction status		COR 95% CI	*P*-value
		Satisfied	Dissatisfied		
Age	< 35	138	186	1	
	≥ 35	22	26	0.877 (0.477–1.612)	0.672
Educational status	Illiterate	37	44	1	
	Literate	123	168	1.149 (0.700–1.885)	0.584
Marital status	Single	26	24	1**	
	Marriage	128	182	0.649 (0.357–1.182)	0.158
	Widowed-divorce	6	6	0.923 (0.262–3.255)	0.901
Residence	Rural	45	85	1**	0.017
	Urban	115	127	1.710 (1.101–2.658)	
Occupation	Housewife	77	91	1**	
	Private employee	10	23	0.514 (0.230–1.146)	0.104
	Government employee	38	30	1.497 (0.849–2.639)	0.163
	Merchant	12	17	0.834 (0.375–1.855)	0.657
	Farmer	18	38	0.560 (0.296–1.059)	0.075
	Student	5	13	0.455 (0.155–1.332)	0.151
Monthly income	< 500	49	61	1	
	501–1500	23	42	0.682 (0.362–1.283)	0.265
	1501–2500	31	40	0.965 (0.529–1.760)	0.907
	2501–3500	15	28	0.667 (0.321–1.386)	0.278
	3501–4500	12	10	1.494 (0.596–3.747)	0.392
	> 4501	30	31	1.205 (0.643–2.256)	0.561
Parity	Null	4	10	1**	
	Prim-para	64	96	0.600(0.180–1.996)	0.405
	Multipara	92	106	0.461(0.140–1.519)	0.203
Schedule of surgery	Emergency/unplanned	105	172	1**	0.001
	Elective /planned	55	40	2.252 (1.402–3.619)	
Previous medical Hx	No	143	187	1	
	Yes	17	25	0.870 (0.260–6.575)	0.725
Previous Surgical history	Yes	48	55	1.223 (0.775–1.932)	0.387
	No	112	157	1	
Referred history	Yes	103	155	1**	0.071
	No	57	57	1.505 (0.966–2.345)	
written consent form version	Defer from mother tongue	40	84	1**	0.009
	In mother tongue	109	125	1.831 (1.161–2.889)	
Consent request by	ob-gyn specialist	33	11	4.800 (1.690–13.634)	0.003
	GP or resident	117	185	1.012 (0.444–2.305)	0.978
	Midwives /nurse	10	16	1**	
Timing of consent	the day before the date of surgery	45	20	3.150 (0.891–11.136)	0.075
	on the day of surgery	33	54	0.856 (0.251–2.917)	0.803
	immediately before surgery	77	131	0.823 (0.252–2.682)	0.746
	on the operation table	5	7	1**	
Time spent to decide	< 1 h	147	196	0.923 (0.431–1.979)	0.837
	≥ 1 h	13	16	1	
Time spent to provide informed consent	< 5 min	72	159	1**	
	5–10 min	44	32	3.036 (1.781–5.178)	0.001
	> 10 min	44	21	4.627 (2.566–8.345)	0.001
who sign on the informed consent form	Self	146	206	1**	.017
	Parent/spouse	14	6	3.292 (1.236–8.768)	
Knowledge towards SIC	Poor knowledge	118	169	1**	0.176
	Good knowledge	42	43	1.399 (0.861–2.274)	
Patient to healthcare provider relationship	Poor	47	146	1**	0.001
	Good	113	66	5.319 (3.400–8.320)	

*COR* crude odd ratio 1: Reference category **Candidate variable for multivariate logistic regression

**Table 6:** Multivariable logistic regression of factors associated with patient's satisfaction towards SIC, 2020

Variables	Category	Satisfaction status		COR (95% CI)	AOR (95% CI)
		Satisfied	Dissatisfied		
Residence	Rural	45	85		1
	Urban	115	127	1.710 (1.101–2.658)	2.279 (1.257–4.131)**
Referred history	Yes	103	155		1
	No	57	57	1.505 (0.966–2.345)	1.806 (1.014–3.215)*
Written consent form version	Defer from mother tongue	40	84		1
	With mother tongue	109	125	1.831 (1.161–2.889)	2.572 (1.422–4.655)*
Time spent while providing SIC	< 5 min	72	159		1
	5–10 min	44	32	3.036 (1.781–5.178)	5.726(2.755–11.900)**
	> 10 min	44	21	4.627 (2.566–8.345)	2.129 (0.882–5.141)
Patient to health care providers relationship	Poor	47	146		1
	Good	113	66	5.319 (3.400–8.320)	7.752 (4.402–13.652)**

*COD* crude odd ratio, *AOD* adjusted odd ratio

*Significant at *P*-value at < 0.05

**Significant at *P*-value < 0.01

The results of multivariable logistic regression analysis showed that the respondents who came from urban settings were 2.2 times (AOR: 2.279, 95% CI 1.257–4.131) more likely satisfied than those who came from rural residences. Respondents who had no referred history from other health settings were 1.8 times (AOR: 1.806, 95% CI 1.014–3.215) more likely satisfied with the provision of informed consent compared to those who had referred history from another health facility.

Women who received written informed consent with mother tongue were two times (AOR: 2.572, 95% CI 1.422–4.655) more likely satisfied with informed consent than their counterparts. Patients who had received information about surgical informed consent for 10 min duration were 5 times (AOR: 5.726, 95% CI 2.755–11.900) more likely satisfied with the provision of informed consent than patients who have received the information for less than 5 min. Furthermore, women who had a good patient-health care providers relationship were 7 times (AOR: 7.752, 95% CI 4.402–13.652) more likely satisfied with the informed consent process than-their-counterpart.

## Discussion

This study reported that women's satisfaction with surgical informed consent was 43% (95% CI 38.2% to 48.7%). The finding of this result is almost similar with studies conducted in Egypt and Eritrea which accounted for 48.4% and 45% of patients satisfied with SIC provision respectively [20, 36]. However, it is lower than studies conducted in the Netherlands, Israel, Hawassa-Ethiopia, Rwanda, and Pakistan on patient satisfaction with informed consent which accounted for 80.8%, 80%, 62.1%, 67.4%, and 48.9% respectively [14, 16, 17, 19, 21]. The discrepancy might be due to variations in health care service quality and patient flow rate.

The findings of this study showed that women from urban residences were two times more likely satisfied with the informed consent than respondents who came from rural residences. This was supported by a study conducted in Eritrea; patients who came from urban settings were more satisfied with informed consent than those who came from a rural setting [20]. This might be because urban living populations have a better chance of receiving health care services promptly and they are nearer to hearing health-related information.

According to this study; patients who hadn’t referred history from other health settings were 1.8 times more likely to be satisfied than those who had referred history from another health facility. A possible reason might be that patients who had a referred history from other health facilities have been worried about their case or diseases because their case was beyond the service provided by the local health facility and they came to seek further investigation and intervention. They also had to be anxious and stressed in the new hospital environment and healthcare providers since they came from another hospital.

Moreover, women's satisfaction with surgical informed consent was statistically significant for the language of written informed consent. Women who received written informed consent with mother tongue were twice more likely to be satisfied than their counterparts. This is supported by a study conducted in Switzerland in which the consent form written in a simple layman's language and wording improves patients' ability to understand the information provided and is strongly associated with patient satisfaction [15]. Simply providing information doesn't guarantee that a patient understands what healthcare providers tell them unless we provide the information within mother tongue because they have different levels of understanding [21].

Furthermore, the duration of time spent during provision surgical informed consent was positively associated with women's satisfaction with surgical informed consent. Patients who had received information about surgical informed consent for longer than 10 min were 5 times more likely to be satisfied than patients who received the information for less than 5 min. This is supported by a study conducted in Nigeria revealed that; time spent explaining surgical procedures and the possible outcome could calm patients and reduce their level of anxiety [37]. This might be due to the adequate time provided to women during the informed consent process allowing them to easily understand the information provided. When health care providers explained the proposed surgery with adequate time, the women coped from anxiety and have been satisfied.

Additionally, women's satisfaction with informed consent was significantly associated with the patient to health care provider relationship. According to this study, the respondents who had a good relationship with health care providers were seven times more likely to be satisfied with the informed consent process than their counterparts. This is supported by a study conducted in the Netherland which reported that a better-perceived patient-to-doctor relationship was related to higher patient satisfaction [26]. Similarly, a study conducted in Australia revealed that the proper delivery of patient-centered health care services with effective communication skills resulted in trust between the patient and healthcare provider and improved patient satisfaction [38].

### Strength and Limitation of the study

As strength, new study or investigation for the current study setting and had a high response rate. As a limitation this study was conducted solely on women who underwent obstetrics and gynecology-related procedures, it didn't address major surgery, so it might have a generalizability issue. The social desirability bias is also limitation of this study.

## Conclusion

Women's satisfaction with the provision of surgical informed consent was inadequate. Residence, written consent form version, time spent, applied the recommended component of SIC, and patient to healthcare provider relationship were significant predictors of women's satisfaction with informed consent. Healthcare providers need to emphasize following standard informed consent guidelines and give enough time while the provision surgical informed consent to patients. Moreover, all concerned bodies have to work collaboratively to make proper SIC service and enhance patient satisfaction.

## Data Availability

The datasets used for this study could be available from the corresponding author upon reasonable request.

## Abbreviations

AOR: Adjusted odd ratio
COR: Crude odd ratio
JMC: Jimma medical center
SIC: Surgical informed consent
